# Hepatocellular carcinoma risk-stratification based on ASGR1 in circulating epithelial cells for cancer interception

**DOI:** 10.3389/fmolb.2022.1074277

**Published:** 2022-11-28

**Authors:** Amparo Roa-Colomo, María Ángeles López Garrido, Pilar Molina-Vallejo, Angela Rojas, Mercedes González Sanchez, Violeta Aranda-García, Javier Salmeron, Manuel Romero-Gomez, Jordi Muntane, Javier Padillo, Jose María Alamo, Jose A. Lorente, María José Serrano, M. Carmen Garrido-Navas

**Affiliations:** ^1^ Clinical Medicine and Public Health Doctoral Program, University of Granada, Granada, Spain; ^2^ Gastroenterology and Hepatology Department, San Cecilio University Hospital, Granada, Spain; ^3^ Gastroenterology and Hepatology Department, Virgen De Las Nieves University Hospital, Granada, Spain; ^4^ Genyo-Centro Pfizer-Universidad De Granada-Junta De Andalucía De Genómica e Investigación Oncológica, Granada, Spain; ^5^ Seliver Group, Institute of Biomedicine of Seville (IBiS)/ Hospital Universitario Virgen Del Rocío/CSIC/Universidad De Sevilla, Seville, Spain; ^6^ Spanish Network for Biomedical Research in Hepatic and Digestive Diseases (CIBERehd), Carlos III Health Institute (ISCIII), Madrid, Spain; ^7^ Statistician at Fundación para la Investigación Biosanitaria Andalucía Oriental Alejandro Otero (FIBAO), Hospital Virgen de las Nieves, Granada, Spain; ^8^ Institute of Biomedicine of Seville (IBiS), Hospital University Virgen del Rocío/CSIC/University of Seville, Seville, Spain; ^9^ Department of Medical Physiology and Biophysics, University of Seville, Seville, Spain; ^10^ General and Gastrointestinal Surgery Division, Virgen del Rocío University Hospital, Sevilla, Spain; ^11^ Legal Medicine Department, Medicine School, University of Granada, Granada, Spain; ^12^ Comprehensive Oncology Division, Clinical University Hospital, Virgen de las Nieves-IBS, Granada, Spain; ^13^ Department of Pathological Anatomy, Faculty of Medicine, University of Granada, Granada, Spain

**Keywords:** hepatocellular carcinoma, liver cirrhosis, circulating tumor cells, precision medicine, cancer interception

## Abstract

**Purpose:** Lack of diagnostic and prognostic biomarkers in hepatocellular carcinoma impedes stratifying patients based on their risk of developing cancer. The aim of this study was to evaluate phenotypic and genetic heterogeneity of circulating epithelial cells (CECs) based on asialoglycoprotein receptor 1 (ASGR1) and miR-122-5p expression as potential diagnostic and prognostic tools in patients with hepatocellular carcinoma (HCC) and liver cirrhosis (LC).

**Methods:** Peripheral blood samples were extracted from LC and HCC patients at different disease stages. CECs were isolated using positive immunomagnetic selection. Genetic and phenotypic characterization was validated by double immunocytochemistry for cytokeratin (CK) and ASGR1 or by *in situ* hybridization with miR-122-5p and CECs were visualized by confocal microscopy.

**Results:** The presence of CECs increased HCC risk by 2.58-fold, however, this was only significant for patients with previous LC (*p* = 0.028) and not for those without prior LC (*p* = 0.23). Furthermore, the number of CECs lacking ASGR1 expression correlated significantly with HCC incidence and absence of miR-122-5p expression (*p* = 0.014; *r* = 0.23). Finally, overall survival was significantly greater for patients at earlier cancer stages (*p* = 0.018), but this difference was only maintained in the group with the presence of CECs (*p* = 0.021) whereas progression-free survival was influenced by the absence of ASGR1 expression.

**Conclusion:** Identification and characterization of CECs by ASGR1 and/or miR-122-5p expression may be used as a risk-stratification tool in LC patients, as it was shown to be an independent prognostic and risk-stratification marker in LC and early disease stage HCC patients.

## 1 Introduction

Liver cancer affected more than 900,000 individuals worldwide in 2020 and the estimated incidence is expected to rise to 1.4 million individuals in 2040 (Cancer Tomorrow, 2022). Hepatocellular carcinoma (HCC) is the most common liver cancer, accounting for 75%–85% of all primary liver tumors (Sung et al., 2021). The risk of HCC is known to be increased by external factors (such as excessive alcohol consumption, viral infections, aflatoxin exposure) inducing liver inflammation and hepatic fibrosis progression; however, the impact of internal factors (beyond fatty liver disease) increasing the risk of developing HCC is still poorly studied (Forner et al., 2018). The known HCC risk factors promote liver cirrhosis (LC), which is usually a prior finding in 80% HCC patients (Llovet et al., 2021). In fact, 2%–5% of cirrhotic patients will develop HCC annually (Fateen and Ryder, 2017), so surveillance by liver ultrasound screening is performed in LC for early diagnosis of HCC (Forner et al., 2018; Reig et al., 2021). Unfortunately, curative treatments (based on surgery or liver transplant) are available in less than 60% of cases due to late diagnosis (Cadier et al., 2017). There is an absence of tissue biopsies because diagnosis of HCC in LC patients is mainly based on dynamic imaging tests, what reduces the ability to molecularly characterize the tumor (Reig et al., 2021). In addition to imaging tests, serum markers such as alpha-fetoprotein (AFP) that is linked to the evolutionary stage of the tumor, have been used for diagnosis and prognosis of HCC (Fateen and Ryder, 2017). However, the fact that 1) AFP may be elevated in chronic hepatitis without HCC (Force et al., 2022) 2) many small-sized HCCs have normal AFP levels (Carr et al., 2018), 3) 30% of HCC patients have normal AFP levels at diagnosis (Lee et al., 2019) and 4) high AFP levels were found in HCC-free individuals (Kobeisy et al., 2012), highlights the lack of sensitivity and specificity of this marker (Bialecki and di Bisceglie, 2005). Although AFP levels greater than 400 ng/ml are considered diagnostic of HCC as well as a marker of bad prognosis marker (Bialecki and di Bisceglie, 2005), it is not recommended to use AFP as a sole marker for HCC surveillance (Hanif et al., 2022) and the combination with other blood-based biomarkers is suggested to improve HCC diagnosis (Wang and Zhang, 2020).

Liquid biopsies have the potential to improve sensitivity/specificity as they are non-invasive, represent better the tumor heterogeneity and can be used to monitor disease evolution. The most widely studied type of liquid biopsy with demonstrated clinical utility are Circulating Tumor Cells or CTCs. The evaluation of CTCs in terms of occurrence, cell count and phenotypic characterization has demonstrated its prognostic value in several solid tumors, including lung (Bayarri-Lara et al., 2016), (de Miguel-Pérez et al., 2019), breast (Nadal et al., 2013), (Ye et al., 2019) and colorectal (Delgado-Ureña et al., 2018) cancers. With respect to HCC, several meta-analyses demonstrated the diagnostic and prognostic utility of detecting CTCs (Fan et al., 2015), (Sun et al., 2017), (Cui et al., 2020), although different isolation and detection technologies may undermine their clinical utility. Isolation methodologies based on EpCAM such as CellSearch^®^ are widely used, although elevated EpCAM expression levels were only found in metastatic HCC lesions compared to primary and vascular invaded tumor (Tsuchiya et al., 2019). Furthermore, elevated EpCAM expression levels were also linked to poor prognosis (Shimada et al., 2019). Thus, using alternative methodologies for CTC isolation, such as those based on size (Wang et al., 2018), (Qi et al., 2018) or including other biomarkers such as glypican-3 (Court et al., 2018) among others could overcome CellSearch^®^ limitations. However, Wang et al. (2018) reported lack of correlation between CTCs and recurrence after liver transplantation even though CTCs were detected in more than two-thirds of HCC patients. This suggests that not only improvements for CTC isolation are needed, but also that CTC characterization might be useful for diagnostic and prognostic purposes. Furthermore, presence of Circulating Epithelial Cells (CECs) in patients with pre-malignant diseases such as chronic obstructive pulmonary disease (COPD) had prognostic and diagnostic value (Romero-Palacios et al., 2019) in the context of Cancer Interception (Serrano et al., 2020), although it has been poorly studied in the context of liver cirrhosis (Chen et al., 2020). In fact, in most studies including LC patients, CTCs/CECs were not detected (Vona et al., 2004) or reported percentages were very low (Takahashi et al., 2021), possibly due to either patient selection or isolation methodology.

One liver-specific biomarker used to isolate CTCs from HCC patients is the asialoglycoprotein receptor 1 (ASGR1) (Li et al., 2014; Court et al., 2018), that represents the human lectin subunit 1 of the asialoglycoprotein receptor. ASGR1 heterodimerizes with ASGR2 (human lectin subunit 2) to produce a transmembrane protein primarily present in sinusoidal and basolateral hepatocellular membranes. The main role of ASGR1 is to bind galactosyl residues, facilitating glycoproteins turnover (Muramatsu, 2007). In fact, it was recently studied in the context of cardiovascular diseases (Xie et al., 2021; Wang et al., 2022a) demonstrating that its inhibition reduces hypercholesterolemia and atherosclerosis (Xie et al., 2021) by increasing cholesterol excretion (Wang et al., 2022a). In the context of liver cancer, the role of ASGR1 as tumor suppressor was first suggested *via* its interaction with LASS2 (longevity assurance homolog 2 of yeast LAG1) (Gu et al., 2016) and more recently by its association with DNA methylation (Zhu et al., 2022), confirming the prior findings of loss of ASGR1 expression in HCC tissue (Shi et al., 2013; Witzigmann et al., 2016). Another potential biomarker of HCC is miR-122-5p, a microRNA involved in multiple physiological processes in the liver that was found to suppress cell proliferation and malignant transformation of hepatocytes (Hu et al., 2012). Downregulation of miR-122-5p in HCC patients as well as HCC-derived cell lines was demonstrated together with an inverse correlation with cyclin G1 expression (Gramantieri et al., 2007) and upregulation of miR-122-5p was shown to repress the epithelial to mesenchymal transition (EMT) through the WNT/β-cadherin signaling pathway *via* Snail 1/2 (Jin et al., 2017). In plasma, miR-122-5p together with other four miRNAs were described as a diagnostic tool (Jin et al., 2019) and recently, its prognostic role was demonstrated when survival of HCC patients was greater in those with higher miR-122-5p expression (Wang et al., 2022b).

In this proof-of-concept study, we evaluated the potential diagnostic and prognostic role of circulating epithelial cells (CECs) using an isolation methodology based on immunomagnetic selection with cytokeratins 7/8 (CK) followed by ASGR1 and miR-122-5p characterization in a cohort of patients suffering from LC, with and without subsequent HCC.

## 2 Material and methods

### 2.1 Study design and sample collection

This prospective cohort study included 113 patients aged between 32 and 86 years suffering from hepatocellular carcinoma (HCC, *N* = 71) or cancer-free liver cirrhosis (LC; *N* = 42). Inclusion criterion for cirrhotic patients was diagnosis of LC by dynamic imaging tests (computerized axial tomography and/or magnetic resonance imaging). Tumor staging for HCC patients followed the BCLC (Barcelona Clinic Liver Cancer) classification (Forner et al., 2018). All patients were aged over 18 and had no other liver disease beyond liver cirrhosis and/or HCC (exclusion criteria). For analytical purposes, the HCC cohort was divided into those diagnosed at an earlier stage (eHCC = BCLC 0-A; *N* = 30) and those diagnosed at a later stage (aHCC = BCLC B-C-D; *N* = 41). Of the 30 eHCC patients, only 11 were subjected to liver transplant. Patients were recruited between 2017 and 2020 at the Gastroenterology and Hepatology Units of three Andalusian University hospitals: San Cecilio (Granada), Virgen de las Nieves (Granada) and Virgen del Rocio (Seville). Informed consent was obtained from all patients before blood extraction and the study protocol was approved by the Hospital´s Ethics Committee following the ethical guidelines of the 1975 Declaration of Helsinki. Not signing the informed consent and not fulfilling the inclusion criteria were exclusion criteria. Samples were anonymized upon blood collection to ensure patients’ privacy and clinicians involved in the project updated the database for clinical information. Patients’ clinical and pathological characteristics are shown in Table 1.

**TABLE 1:** Risk factors for HCC. Abbreviations are: HCC, hepatocellular carcinoma; n, number of individuals; SD, standard deviation; Dx, diagnosis; INR, international normalized ratio; AFP, alpha-fetoprotein; HCV, hepatitis C virus; CEC, circulating epithelial cells; IQ, interquartile; CK, cytokeratin; ASGR1, asialoglycoprotein receptor 1. For quantitative analyses *t*-student and Mann-Whitney tests were used depending on data normality. For qualitative data, Chi-Square was used except for those cases with less than 20% of the data with lower than 5 expected frequencies, in which case, Fisher test was used. *p* values are: ***p < 0.001 and **p < 0.05. Bold is the mean for each value as a whole.

	HCC-free *N* = 42	HCC-affected *N* = 71	*p* values
Sex [*n* (%)]			<0.001***
Female	16 (38.1%)	7 (9.9%)	
Male	26 (61.9%)	64 (90.1%)	
Age (mean ± SD, years)	**66 ± 9**	**66 ± 10**	0.784
Female	67 ± 9	63 ± 11	
Male	64 ± 9	66 ± 10	
Cirrhosis Dx (mean ± SD, years)	**61 ± 10**	**62 ± 11**	0.529
Female	63 ± 10	63 ± 13	
Male	59 ± 10	62 ± 11	
Bilirubin levels (mean mg/dL)	**0.96 ± 0.38**	**1.70 ± 2.82**	0.034**
Female	0.90 ± 0.44	1.16 ± 1.89	
Male	1.0 ± 0.34	1.76 ± 2.91	
Albumin levels (mean g/dL)	**4.10 ± 0.40**	**3.70 ± 0.60**	<0.001***
Female	3.90 ± 0.40	3.70 ± 0.70	
Male	4.20 ± 0.30	3.70 ± 0.60	
Platelets number (mean 10^6^/L)	**146,524 ± 78,641**	**152,052 ± 103,980**	0.767
Female	151,125 ± 69,214	173,957 ± 172,546	
Male	143,692 ± 85,125	149,656 ± 95,491	
INR (mean ± SD)	**1.13 ± 0.35**	**1.23 ± 0.32**	0.116
Female	1.26 ± 0.54	1.12 ± 0.18	
Male	1.05 ± 0.12	1.25 ± 0.33	
Prothrombin activity (%)	**90 ± 21**	**77 ± 18**	<0.001***
Female	84 ± 27	83 ± 77	
Male	94 ± 16	77 ± 18	
AFP baseline (mean ng/mL)	**6.13 ± 16.65**	**1,111.62 ± 4,685.08**	<0.001***
Female	11.15 ± 27.64	5,333.91 ± 13,330.00	
Male	3.32 ± 1.38	619.03 ± 2,030.34	
Alcohol, n (%)			0.17
Female	4 (33.3%)	4 (14.33%)	
Male	8 (66.7%)	24 (85.7%)	
HCV, *n* (%)			0.045**
Female	7 (35%)	1 (6.3%)	
Male	13 (65%)	15 (93.8%)	
Child Pugh-Turcotte, *n* (%)			<0.0001***
A (5–6)	40 (100)	48 (71.6)	
B (7–9)	0	15 (22.4)	
C (10–15)	0	4 (6.0)	
CEC phenotype, *n* (median; IQ range)			0.044**
CK+/ASGR1+	17 (2; 4)	29 (2; 2)	
CK+/ASGR1−	7 (1; 4)	25 (2; 7)	
Negative	18 (0)	17 (0)	
Total sum of CECs			0.0002***
CK+/ASGR1+	36	48	
CK+/ASGR1−	15	74	

### 2.2 Circulating epithelial cell isolation

Peripheral blood samples (15 ml) were collected in K2-EDTA Vacutainer tubes at the time of diagnosis. Blood samples were enriched in peripheral mononuclear blood cells using gradient centrifugation with Ficoll-Histopaque^®^-1119 and CECs were isolated using the Carcinoma Cell Enrichment Kit (130-060-301, Miltenyi Biotec) based on pan-anti-cytokeratin, as previously described (Nadal et al., 2013). Isolated CECs were then spun down onto Poly-L-lysine-coated glass slides using a cytospin (Hettich) for subsequent phenotypic and genetic characterization (one glass slide was prepared per 7.5 ml peripheral blood).

### 2.3 CECs enumeration and phenotypic characterization by ASGR1 detection

Before isolating and characterizing CECs from patients, antibody specificity for ASGR1 was assessed on the hepatocellular tumor cell line HEPG2 (reference 85011430, lot. 2440) obtained by the Centre of Scientific Instrumentation at the University of Granada. The primary alveolar epithelial cell line hAELVi (InSCREENeX GmbH) was selected as a negative control for ASGR1 expression as stated in the human protein atlas and peripheral blood mononuclear cells (PBMCs) from a healthy donor were used as negative control for both antibodies (Supplementary Figure S1). Both cell lines were tested with STR assay for cell authenticity and for mycoplasma contamination. All experiments were done in duplicates. Subsequently, isolated CECs from patients were enumerated and characterized using double immunocytochemistry with chromogenic staining for cytokeratin (CK) followed by fluorescent detection (for ASGR1). CK detection was done using the Carcinoma Cell Detection Kit (130-060-301, Miltenyi Biotec) as previously described (Nadal et al., 2013). CECs from HCC and LC patients were then blocked with 10% goat serum and 1.5% FcR blocking in cell stain solution for 45 min and incubated with 1/100 diluted anti-ASGR1 rabbit antibody (Atlas Antibodies Cat# HPA012852, Merck) in 1% goat serum overnight. For visualization of ASGR1, 10 μg of goat anti-rabbit Alexa Fluor 633 antibody (A-21070, Thermo Fisher Scientific) were added for 20 min. Finally, slides were mounted using 4 μg of Hoechst 33342 (Thermo Fisher Scientific) and SlowFade™ Gold Antifade Mounting Media (S36936, Thermo Fisher Scientific). Cell enumeration and characterization was performed in a laser confocal microscope (Zeiss LSM 710) and pictures were taken using a ×60 oil objective. CECs were reported as either CEC^CK+/ASGR1+^ (for positive ASGR1 staining) or CEC^CK+/ASGR1−^ (for negative ASGR1 staining).

### 2.4 CECs genetic characterization by immunoFISH

As a proof-of-concept, we selected 5 individuals positive for CECs belonging to each patient cohort (5 LC, 5 eHCC and 5 aHCC) and a second glass slide for each patient allowed genetic characterization by immunoFISH. This procedure was not performed in all patients due to: availability of enough sample, difficulties of the combination of chromogenic staining with fluorescence detection as well as financial reasons. In patients positive for CECs, *fluorescence in situ hybridization* (FISH) for the miR-122-5p in combination with immunofluorescence with cytokeratin was performed to determine liver-origin of CECs. Samples were treated with filtered 0.1% pepsin (Merck) in 10 mM HCL during 1 min at 37°C, washed twice with PBS and then hybridized for 1 h at 57°C with miRCURY LNA miRNA Detection Probe for miR-122-5p (Qiagen, Cat#339453) according to the manufacturer’s protocol. After increasing stringency washes with SSC samples were blocked (PBS 1x, 0.1% Tween-20, 2% goat serum, 1% BSA) and incubated with anti-digoxigenin antibody (Roche, Cat#11093274910) for 1 h at room temperature. After several PBS-Tween washes, samples were stained using the pre-filtered substrate FastRed for 1.5 h at 30°C. Staining was terminated by addition of KTBT buffer and subsequently, immunofluorescence for cytokeratin-FITC was performed. Supplementary Figure S2 shows internal controls for specificity of probes and methodology.

### 2.5 Statistical analysis

Descriptive analysis of variables was performed using SPSS, calculating measures of central trend and dispersion for the numerical variables; absolute frequencies and percentages for qualitative variables were also calculated. Normality of the data was studied with the Shapiro-Wilks test. A bivariate analysis was carried out to analyze possible factors related to the main variables. For numerical variables, the Student’s t test was applied for independent samples or Mann-Whitney in non-parametric cases. For qualitative variables, Pearson’s Chi-square test or Fisher’s exact test were applied. In addition, odds ratio and its 95% confidence interval were calculated for each variable. With those that were statistically significant, a multivariate logistic regression model was proposed to jointly predict which factors influenced tolerance to treatment. The variable selection method was performed by successive steps backwards, eliminating in each step those variables that did not significantly influence the model, applying the likelihood ratio test. To evaluate the goodness of fit of the model, the Hosmer-Lemeshow statistic was calculated and to calculate survival rates, overall survival (OS) and progression-free survival (PFS) were plotted as Kaplan Meier curves. Statistical significance was considered for *p* < 0.05. Graphs were created using GraphPad.

## 3 Results

### 3.1 Clinical and pathological characteristics of the study cohort

Our study cohort included 113 individuals, 42 cancer-free LC patients and 71 HCC patients (of which only 9 did not have prior LC). Univariate analysis of factors associated with HCC included sex, albumin levels and prothrombin activity (*p* < 0.001), bilirubin (*p* = 0.034), etiology including hepatitis virus C (*p* = 0.045) and Child Pugh-Turcotte stage (Table 1). No relevant information was obtained using multivariate analysis (data not shown).

### 3.2 Circulating epithelial cells characterization in LC and HCC patients

**FIGURE 1:**
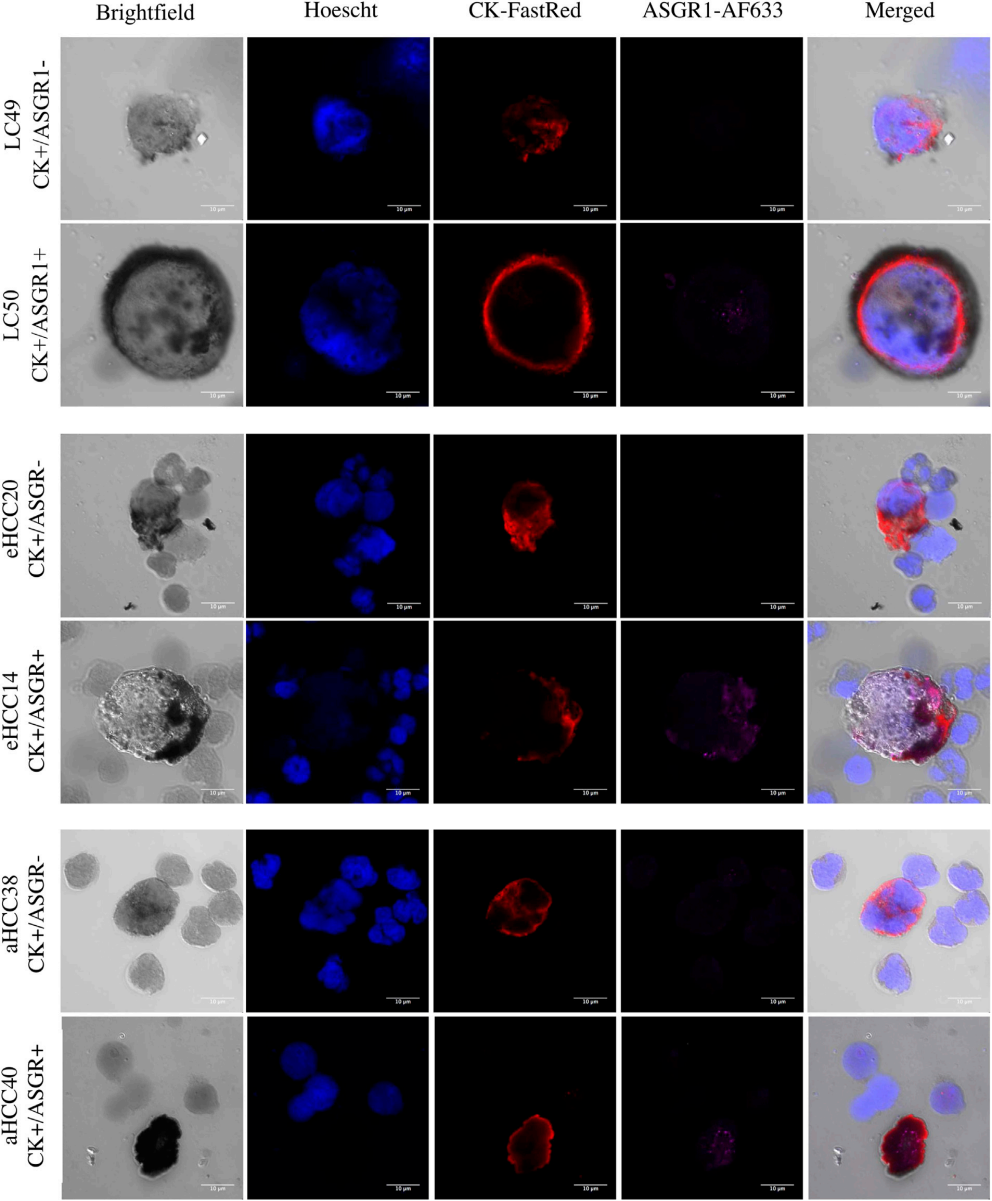
CECs heterogeneity in patients with liver cirrhosis or HCC. Heterogeneity is shown with respect to Cytokeratin (CK) and ASGR1 expression in two LC (liver cirrhosis) patients (top), two eHCC (early HCC) patients (middle) and two aHCC (advanced HCC) patients (bottom). In each case, one CEC positive for the two markers (CK and ASGR1) and one CEC negative for ASGR1 are shown. Hoechst is used as nuclear staining.

CECs were detected in 79 patients (69.9%) with significantly greater frequencies in HCC (54/71; 76.1%) than in HCC-free patients (24/42; 57.1%) (*p* = 0.023). Phenotypic heterogeneity of CECs was identified both intra and inter individual based on ASGR1. Particularly, two phenotypes were identified: ASGR1 positive (CK+/ASGR1+) and negative (CK+/ASGR1−), with varying intensities and sizes (Figure 1).

**FIGURE 2:**
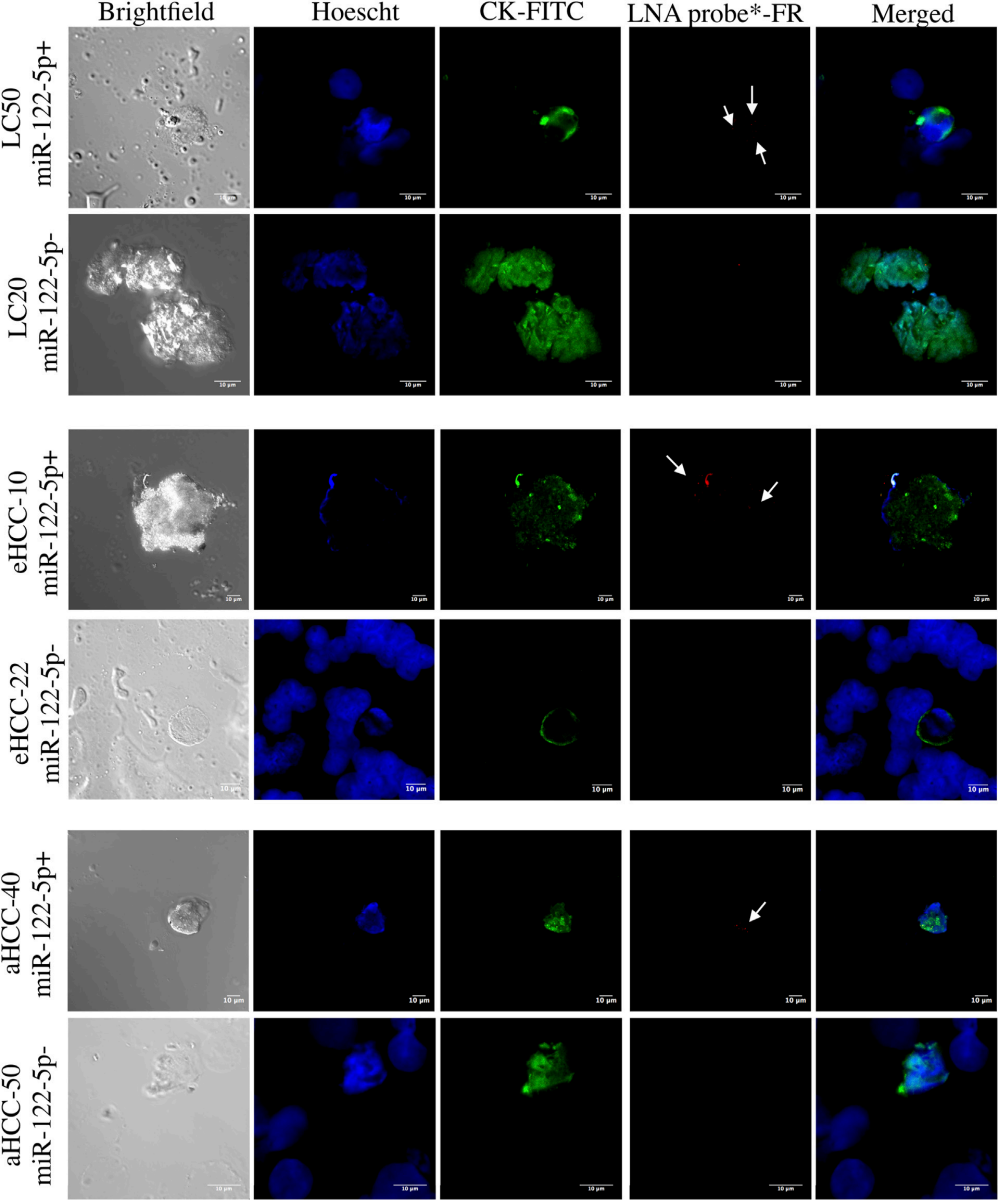
Confocal microscopy of immunoFISH for miR-122-5p in patients with liver cirrhosis or HCC. Cytokeratin (CK) is shown in green (FITC) and miR122-5p probe is shown in red. Data from two patients for each group: liver cirrhosis (LC; top), early HCC (eHCC; middle) and advanced HCC (aHCC; bottom) are shown. For each patient group, a positive miR-122-5p signal (coinciding with positive ASGR1 staining) and a negative miR-122-5p signal (coinciding with negative ASGR1 staining) are shown.

No significant differences were observed between the ASGR1 positive (46/78; 58.9%) and the ASGR1 negative (32/78; 41.1%) phenotype (*p* = 0.33), being ASGR1 expression more prevalent in cirrhotic (17/24; 70.8%) than in HCC (29/54; 53.7%) patients. Importantly, all CECs with the ASGR1 positive phenotype also showed positivity for the miR-122-5p liver-specific marker (Figure 2, rows 1,3 and 5), demonstrating their liver origin. In contrast, absence of miR-122-5p was observed in all patients with CECs showing ASGR1 negative staining (Figure 2, rows 2,4 and 6).

### 3.3 Correlation of CEC phenotypes with clinical and pathological data

**FIGURE 3:**
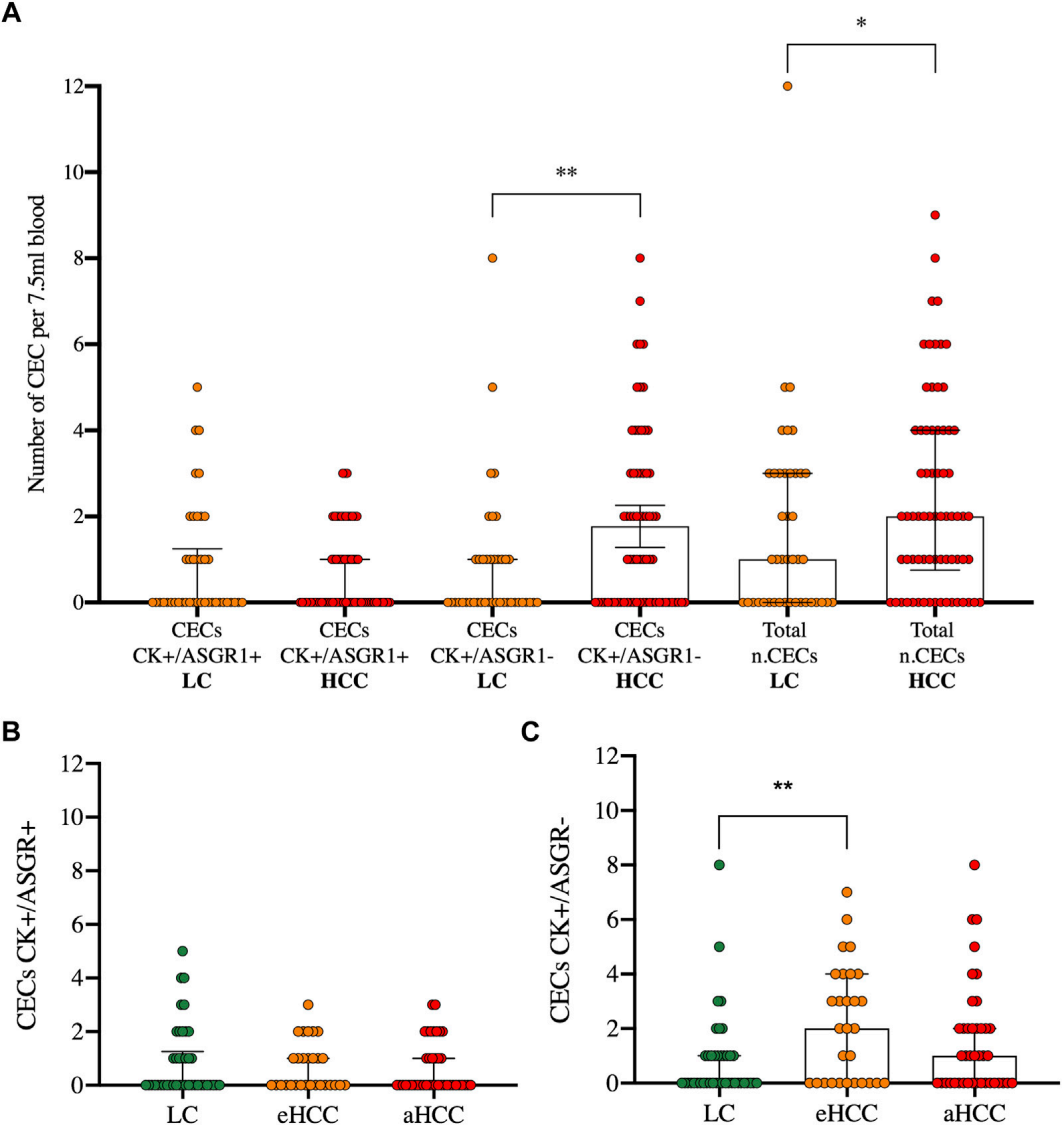
CECs enumeration in blood in patients with liver cirrhosis or HCC. CECs are characterized by either double staining (CK+/ASGR1+) or single staining (CK+/ASGR1-) in liver cirrhosis (LC) and hepatocellular carcinoma (HCC) patients **(A)**. The latter are divided into early (eHCC) and advanced (aHCC) disease for both, double **(B)** and single staining **(C)**. *p* values for Kruskal Wallis test and multiple comparisons are:**p = 0.0096 and *p = 0.035 **(A)** and **p = 0.0051 **(C)**.

The amount of CEC^CK+/ASGR1-^ was significantly greater in HCC than in HCC-free patients (*p* = 0.0096), significantly increasing the total CEC count in HCC compared to LC patients (*p* = 0.035) (Figure 3A). Furthermore, when accounting for disease stage: early (BCLC 0-A) or advanced (BCLC B-C-D), no differences were observed for the ASGR1 positive population (Figure 3B). However, Kruskal-Wallis tests revealed that the number of CECs with absence of ASGR1 expression was significantly different among disease stages (*p* = 0.017). There was a significant increase of CEC^CK+/ASGR1−^ between LC and eHCC (*p* = 0.0051) but not between LC and aHCC (*p* = 0.08) or eHCC and aHCC (*p* = 0.23) (Figure 3C).

**FIGURE 4:**
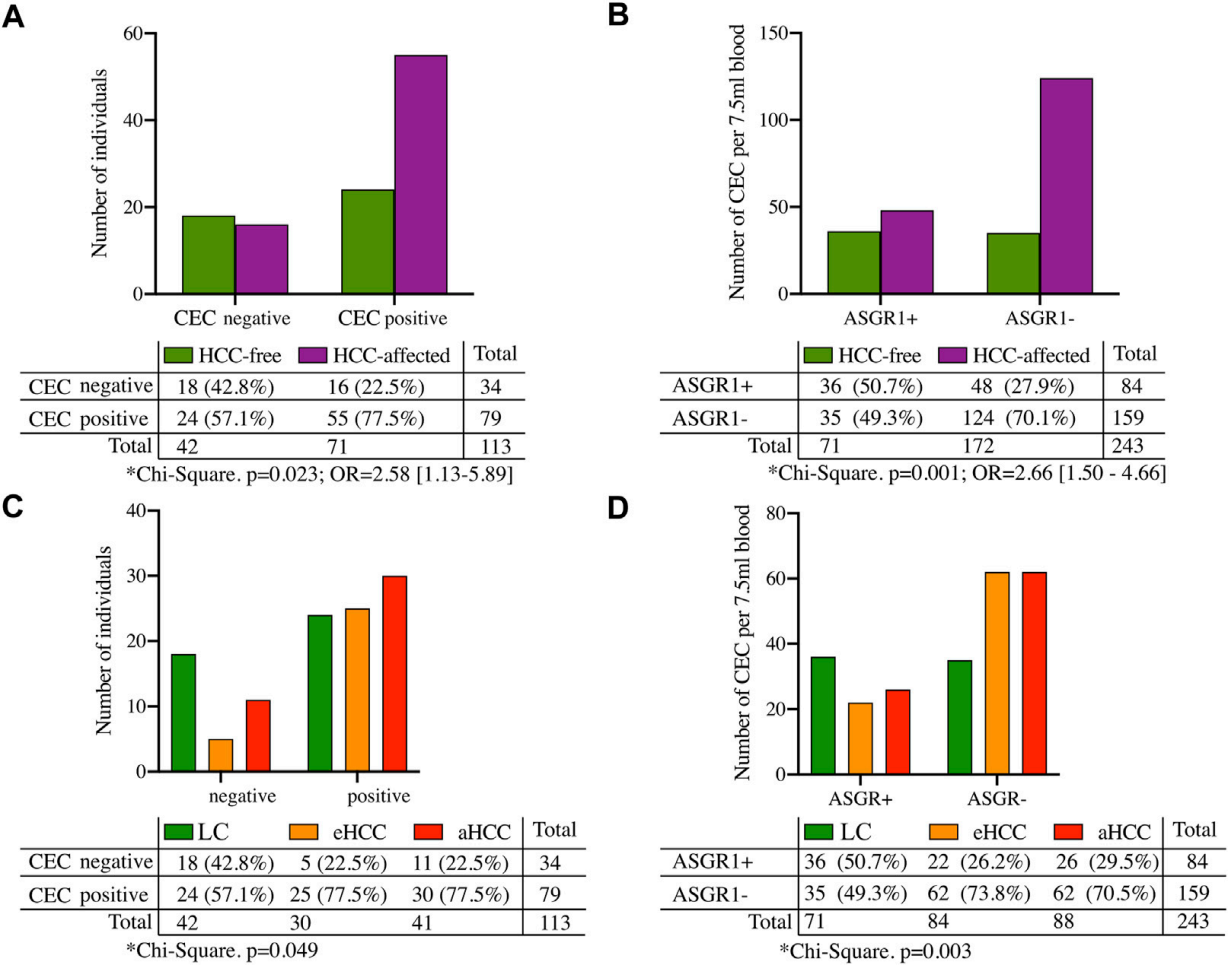
Risk factors for HCC. **(A)** Shows presence/absence of CECs and **(B)** shows number of CECs expressing (or not) ASGR1 in liver cirrhosis (LC) or hepatocellular carcinoma patients (HCC). **(C,D)** show same information as in **(A,B)** respectively, but dividing HCC individuals by either early (eHCC) or advanced (aHCC) disease stages.

Our results suggest that both presence of CECs and absence of ASGR1 expression in CECs are risk biomarkers of HCC. In fact, presence of CECs increased HCC risk by 2.58 -fold (*p* = 0.023) (Figure 4A) and the number of CECs lacking ASGR1 expression significantly (*p* = 0.001) increased risk of developing HCC by 2.66-fold (Figure 4B). A significantly greater proportion of aHCC and eHCC patients had CECs (including ASGR+ and ASGR-) compared with LC (*p* = 0.049) (Figure 4C) and this increase came with a significant reduction on the number of CECs expressing ASGR1 in HCC compared to LC (*p* = 0.003) (Figure 4D).

**FIGURE 5:**
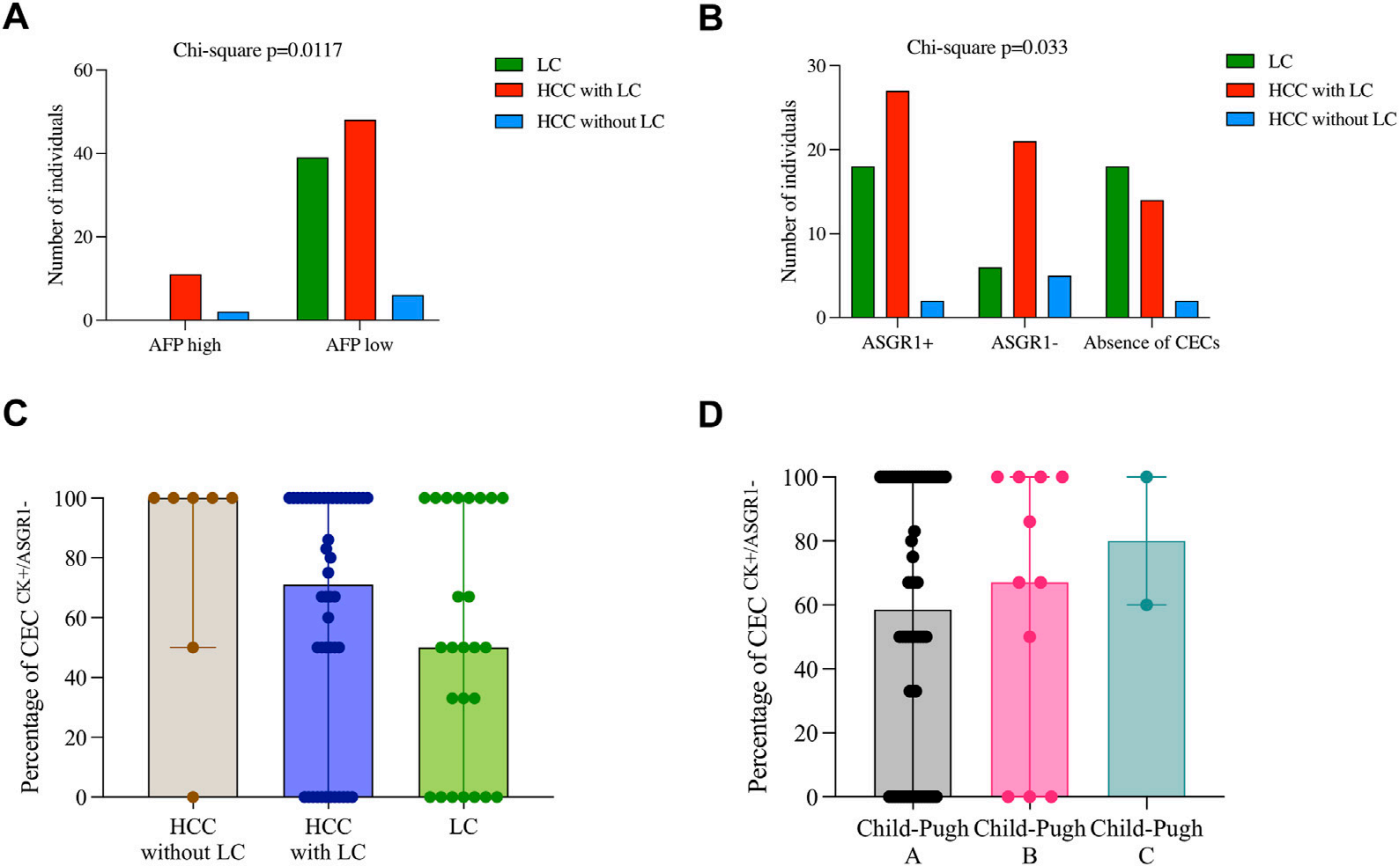
Relationship between CEC characterization and clinical and pathological features in patients with liver cirrhosis or HCC. **(A)** Shows AFP levels divided by high (>400 ng/ml) and low (<400 ng/ml) for three groups of patients: LC = liver cirrhosis; HCC of cirrhosis etiology and HCC of other a etiology. **(B)** Shows frequency distribution of CECs for the same groups of patients. **(C,D)** Represent percentages of CECs with lack of ASGR1 expression for either the three previous group of patients **(C)** or Child-Pugh scores **(D)**. *p* values for Chi-Square tests are shown for graphs **(A,B)**. No significant differences are shown in C and D for Kruskal Wallis tests.

When pathological AFP levels were considered, 13/13 patients (100%) with AFP greater than 400 ng/ml showed HCC (11 of which had prior LC). However, there were a significant (*p* = 0.0117) proportion of individuals with lower AFP levels (54/93; 58.1%) that also developed HCC, highlighting the poor performance of AFP for identifying high-risk individuals (Figure 5A). Interestingly, there was a significant difference between ASGR1 expression when analyzing liver cirrhosis and cancer occurrence together (*p* = 0.033). Thus, of the total of individuals with CECs negative for ASGR1 expression, the majority were HCC patients with prior LC (65.6%; 21/32), while there was no difference between cancer-free LC (18.8%; 6/32) and HCC without prior LC (5/32; 15.6%). Contrarily, positive expression of ASGR in CECs was more frequent in LC (42.9%; 18/42) than in HCC without prior LC (22.2%; 2/9), suggesting that LC patients (independently on their cancer status) were more likely to have ASGR1 positive CECs (Figure 5B). In fact, HCC patients without previous LC had the greatest percentage of CECs without ASGR1 expression (median 100%) compared with HCC with LC (median 71.0%) and HCC-free LC (median 51%) patients although this difference was not significantly different (*p* = 0.21) (Figure 5C). Furthermore, we found that the percentage of CECs without ASGR1 expression increased upon liver dysfunction (Child Pugh-Turcotte stage) (Figure 5D), although this increase was not significant (*p* = 0.74). Presence of CECs without ASGR1 expression significantly correlated with cancer occurrence (*p* = 0.012). Indeed, ASGR1 expression in CECs negatively correlated with INR (*p* = 0.025).

**FIGURE 6:**
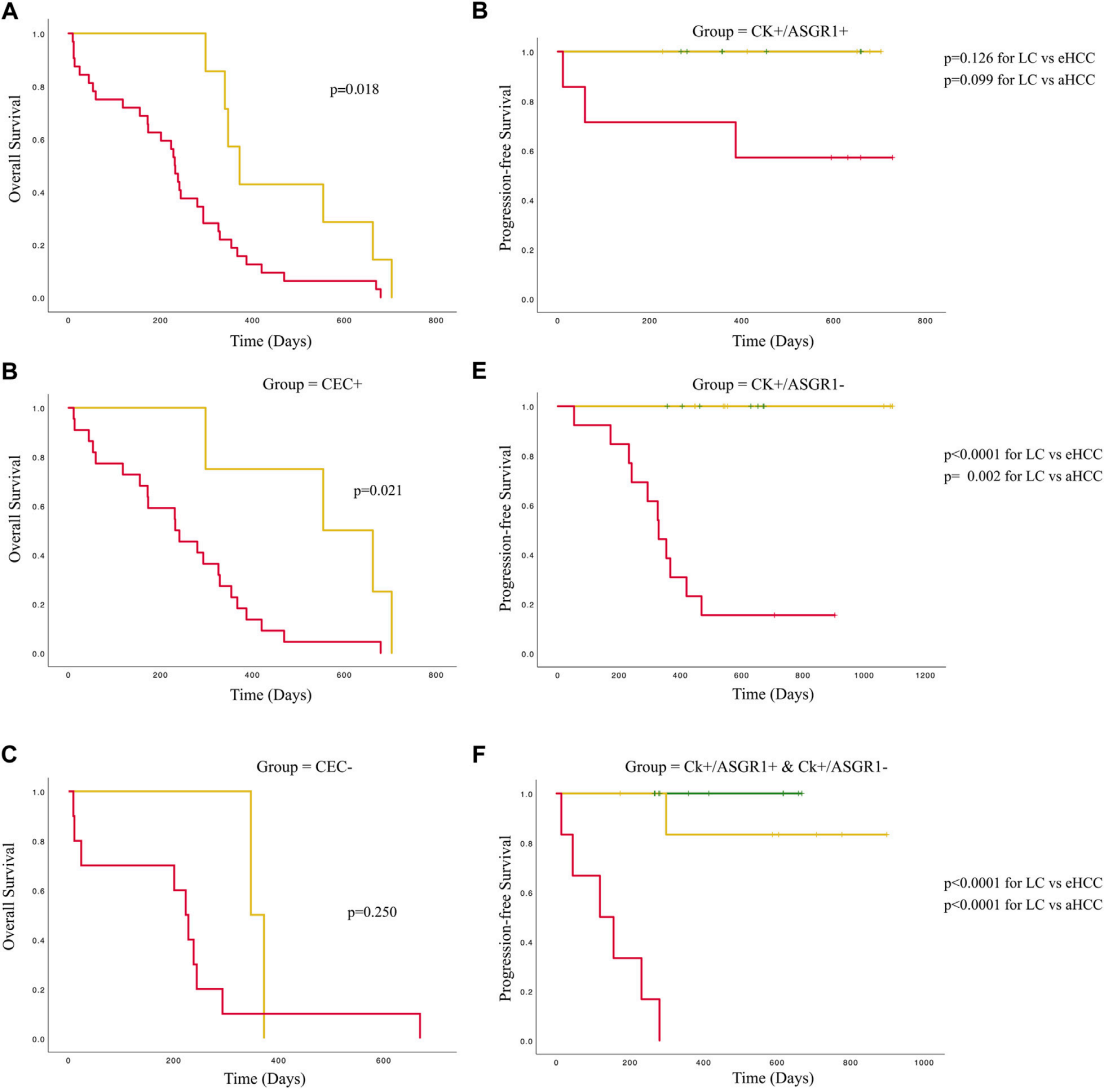
Survival curves for patients with liver cirrhosis or HCC accounting for CEC numbers and phenotype. Left part of the figure represents overall survival curves for the whole population **(A)**, only for those positive for CECs **(B)** and for those negative for CECs **(C)**. Colors are Grey = eHCC and Black: aHCC. Right part of the figure represents progression-free survival for patients with all CECs positive for ASGR1 **(D)**, patients with all CECs negative for ASGR1 **(E)** and patients with heterogeneous CEC phenotypes **(F)**. Colors are: Dotted Grey = liver cirrhosis; Grey eHCC and Black: aHCC.

Finally, as expected, early HCC patients showed significantly greater overall survival (OS) than advanced HCC (*p* = 0.018) (Figure 6A). However, this difference was observed only in patients showing CECs (Figure 6B; *p* = 0.021) and not in patients without CECs (Figure 6C; *p* = 0.250). Furthermore, a significantly different progression-free survival (PFS) was observed considering the two CEC phenotypes. While no significant differences were observed for CECs positive for ASGR1 (Figure 6D; *p* = 0.126, *p* = 0.099 for LC compared to eHCC or aHCC, respectively), significant PFS were observed for CECs negative for ASGR1 expression (Figure 6E; *p* < 0.0001, *p* = 0.002 for LC compared to eHCC or aHCC, respectively). Thus, PFS was significantly lower in aHCC and eHCC patients compared to LC when considering the CEC phenotype (Figure 6F; *p* < 0.0001).

## 4 Discussion

In this study we have demonstrated the suitability of two liver-specific biomarkers (ASGR1 and miR-122-5p) for the characterization of circulating epithelial cells (CECs) from liver cirrhosis (LC) and hepatocellular carcinoma (HCC) patients as prognostic biomarkers. This proof-of-concept study demonstrates that phenotypic characterization of CECs with either ASGR1 or miR-122-5p may allow risk-stratification in patients at earlier disease stages, which is the principal basis of precision medicine.

Currently there is a lack of diagnostic and risk-stratification tools for HCC. Evaluation of circulating tumor cells (CTCs) in advanced stages is a prognostic marker for high risk of metastasis (Wang et al., 2018) and presence of CTCs after surgery or resection is a prognostic marker for PFS in HCC patients (Cui et al., 2020); however, there are some limitations of CTC evaluation, particularly in early and pre-tumoral disease stages. Lu-Nan, Qi et al. (2018) identified CTCs in more than half of the early-stage (BCLC 0-A) HCC patients suggesting it may be a remarkably useful tool for cancer interception (Serrano et al., 2020). However, there are currently only very few studies addressing identification and characterization of CECs in pre-tumoral diseases such as LC (Chen et al., 2020).

Expression of ASGR1 preferentially in the sinusoidal and basolateral hepatocellular membranes makes this protein an important biomarker for HCC. In fact, it has been explored in the context of targeted therapies for HCC (Chen et al., 2017), (Kim et al., 2019), (Nair et al., 2019). Other roles of ASGR1 such as hepatitis C virus binding allowing viral infection (Saunier et al., 2003) or metastasis promotion by interaction with lectins in the tumor microenvironment through the EGFR-ERK pathway (Ueno et al., 2011) are also described in relation with HCC induction. Interestingly, hepatocytes expressing low levels of ASGR1 were characterized as progenitor-like cells with higher levels of EGFR, β1 and α6 integrins expression (Ise et al., 2004). Furthermore, variable ASGR1 expression was observed between tumor stages. For instance, Shi et al. (2013) demonstrated that expression of ASGR1 (based on H-scores) in normal adjacent tissue was comparable to that on hepatic cirrhosis and early HCC (grade I or well differentiated) using tissue microarrays, although its expression decreased significantly with increasing tumor stage. This suggested that ASGR1 could potentially be used as an early indicator of the disease status. Likewise, Witzigmann et al. (2016) showed lower levels of ASGR1 mRNA in HCC compared to its adjacent normal tissue, as well as a reduction of mRNA according to increasing HCC stages. Furthermore, a decreased ASGR1 mRNA expression was observed in metastatic or highly proliferative tumors (defined by Ki69 positivity) (Witzigmann et al., 2016) and overexpression of ASGR1 was shown to inhibit cell migration and invasivity potential both *in vitro* and *in vivo* (Gu et al., 2016), suppressing metastasis and serving a prognostic biomarker.

Together with ASGR, miR-122-5p is another liver-specific biomarker which tumor suppressor role has also been suggested in HCC (Gramantieri et al., 2007; Jin et al., 2017). In fact, miR-122-5p is one of the best diagnostic markers for HCC being usually elevated in circulation of HCC patients (Jin et al., 2019); however, its overexpression in circulation was not correlated with that in tissue, which levels were found to be downregulated in HCC (Gramantieri et al., 2007). Intra-tumor heterogeneity following microenvironment stress cannot be characterized by cytokeratin positive CEC counts alone. Thus, phenotypic characterization with tissue-specific biomarkers, such as ASGR1 or miR-122-5p, may be of great importance. In fact, we identified distinct CEC subpopulations based on ASGR1/miR-122-5p staining within and between patients. Interestingly, a perfect positive correlation was found between the two markers suggesting that both could become excellent prognostic biomarkers for HCC.

Our results show that both the presence of CECs and the absence of ASGR1 expression in CECs are risk factors of developing HCC. Furthermore, ASGR1 staining has been shown to be positively correlated with miR-122-5p expression, suggesting that CECs expressing both markers originated from hepatocytes; contrarily, absence of the two biomarkers suggests a more dedifferentiated state, potentially identifying more aggressive phenotypes. These data agree with those presented by Shi et al. (2013) and Witzigmann et al. (2016), who have shown decreased ASGR1 expression levels in HCC tumor tissues, both at the mRNA and protein level as well as works from Hu et al. (2012) who showed a decreased miR-122-5p expression in HCC compared to controls.

Despite its main expression in liver, ASGR1 was also shown to be expressed in other cell types such as colon (Fang et al., 2009) or peripheral blood mononuclear cells (PBMCs) (Harris et al., 2012), albeit at lower levels compared with hepatocytes. We have also observed some level of ASGR1 expression in PBMCs of some individuals (data not shown); however, any significance of such differences among patients needs to be further investigated. One of the described roles of ASGR1 is the clearance of platelets and other prothrombotic blood components (Sørensen et al., 2009). Therefore, its presence in PBMCs might arise because of this blood hemostasis process. In fact, we showed significant correlations between ASGR1 expression in CECs and INR (*p* = 0.025) as well as prothrombin activity (*p* = 0.006), validating the role of ASGR1 maintaining blood homeostasis. Furthermore, the percentage of ASGR1 expression in CECs decreased with Child Pugh-Turcotte stage suggesting it may be used as diagnostic and prognostic biomarker in earlier disease stages as an outstanding cancer interception tool (Serrano et al., 2020). Thus, our data highlights the clinical utility of characterizing CECs using ASGR1/miR-122-5p isolated from patients with chronic liver cirrhosis or HCC to identify potentially more aggressive phenotypes (loss of ASGR1/miR-122-5p) serving as prognostic tool. A larger clinical trial in which clinical utility of these two biomarkers is assessed, might allow to update the current clinical management guidelines for HCC. Incorporation of liquid biopsy based on CTC isolation and characterization using ASGR1 and/or miR-122-5p might become a non-invasive strategy for risk stratification, improving early identification of malignancy in liver cirrhotic patients.

We understand that the cohort size might impact on our ability to reach good diagnostic power for CECs and ASGR1 as biomarkers, although similar (Qi et al., 2018) and even smaller cohort sizes (Vona et al., 2004; Wang et al., 2018; Takahashi et al., 2021) have already been used by other authors. Also, we acknowledge that detection of miR-122-5p on CECs should have been performed in all samples positive for CECs; however due to funding constraints and difficulties of the methodology, we only assessed as a proof-of-concept this biomarker in a small cohort of patients. We are currently working to increase cohort size and to characterize CECs using a more comprehensive biomarker panel including ASGR1 and miR-122-5p simultaneously using our recently developed protocol (Ruiz-Rodríguez et al., 2021) to improve statistical power and to demonstrate the diagnostic utility of characterizing CECs for LC and HCC patients.

## 5 Conclusion

Circulating epithelial cells (CECs) may prove to become a very useful prognostic biomarker for the identification of individuals at risk of developing hepatocellular carcinoma (HCC). Both the presence of CECs and the lack of ASGR1/miR-122-5p expression in CECs were linked with HCC incidence and poorer disease outcomes, highlighting their potential as predictive biomarkers.

## Data Availability

The original contributions presented in the study are included in the article/Supplementary Material, further inquiries can be directed to the corresponding authors.
