# Supplementary material for: Hepatocellular carcinoma risk-stratification based on ASGR1 in circulating epithelial cells for cancer interception
Source: Front Mol Biosci. 2022 Nov 28;9:1074277. doi: 10.3389/fmolb.2022.1074277 (PMC9742249; doi:10.3389/fmolb.2022.1074277)
Supplement: Supplementary file 1 [file Image2.pdf]

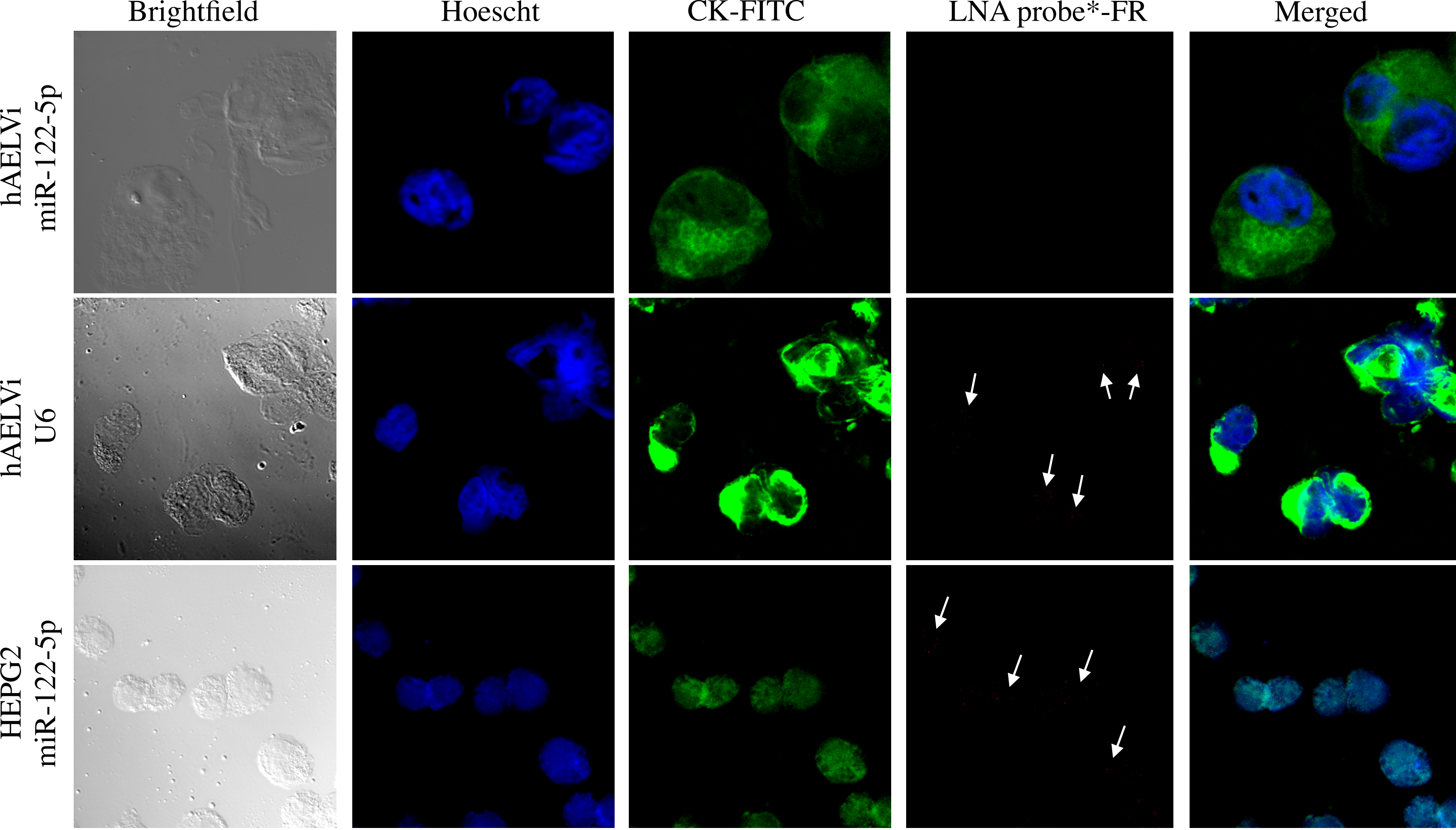

Supplementary figure 2: ImmunoFISH control experiments using LNA probes.

The alveolar epithelial cells hAELVi were used as negative control for LNA-miR-122-5p and as positive for the control LNA-U6 probes visualized using fast red (FR). Cytokeratin (CK) was stained with FITC (green). The hepatocellular carcinoma cell line (HEPG2) shows positive staining of both markers, CK and miR-122-5p whereas hAELVi shows negative staining for miR-122-5p. Hoescht was used as nuclear staining. *LNA probes are shown in the y-axis of the figure as they are different for each experiment.
