# Supplementary material for: Hepatocellular carcinoma risk-stratification based on ASGR1 in circulating epithelial cells for cancer interception
Source: Front Mol Biosci. 2022 Nov 28;9:1074277. doi: 10.3389/fmolb.2022.1074277 (PMC9742249; doi:10.3389/fmolb.2022.1074277)
Supplement: Supplementary file 2 [file Image1.pdf]

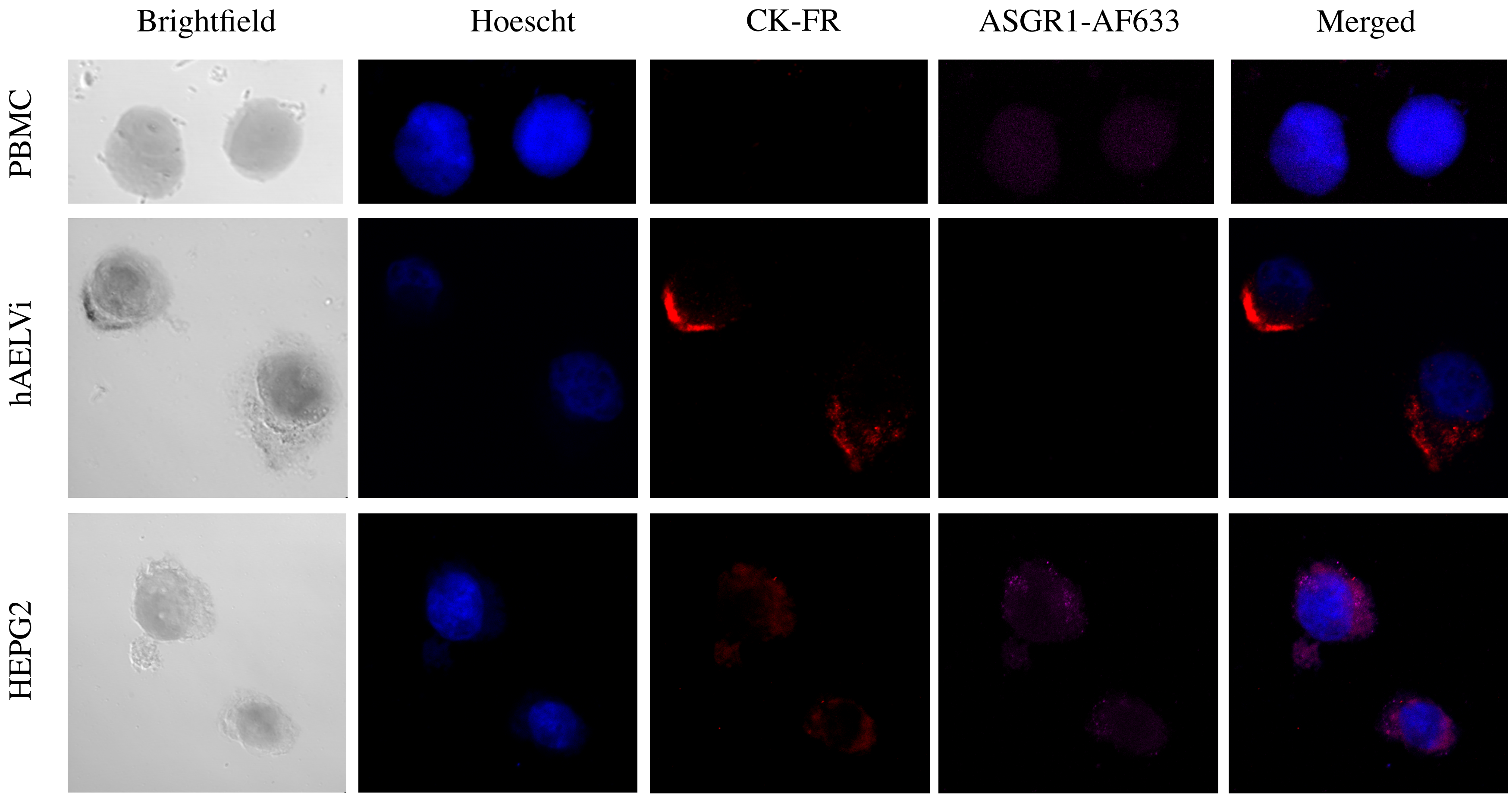


**Supplementary figure 1: Specificity assay of anti-ASGR1 antibody.**

PBMCs from healthy individuals were used as negative controls for both antibodies. The alveolar epithelial cell line hAELVi was used as negative control for ASGR1 antibody staining (with Alexa Fluor 633) and positive for the epithelial marker CK (visualized using FR: fast red). The hepatocellular carcinoma cell line (HEPG2) shows positive staining of both markers, CK and ASGR1. Hoescht was used as nuclear staining.
